# Rhamnogalacturonan-I Based Microcapsules for Targeted Drug Release

**DOI:** 10.1371/journal.pone.0168050

**Published:** 2016-12-19

**Authors:** Anna J. Svagan, Anja Kusic, Cristian De Gobba, Flemming H. Larsen, Philip Sassene, Qi Zhou, Marco van de Weert, Anette Mullertz, Bodil Jørgensen, Peter Ulvskov

**Affiliations:** 1 Department of Pharmacy, University of Copenhagen, Copenhagen, Denmark; 2 Department of Food Science, University of Copenhagen, Copenhagen, Denmark; 3 School of Biotechnology, Royal Institute of Technology (KTH), Stockholm, Sweden; 4 Department of Plant and Environmental Sciences, University of Copenhagen, Copenhagen, Denmark; Iowa State University, UNITED STATES

## Abstract

Drug targeting to the colon via the oral administration route for local treatment of e.g. inflammatory bowel disease and colonic cancer has several advantages such as needle-free administration and low infection risk. A new source for delivery is plant-polysaccharide based delivery platforms such as Rhamnogalacturonan-I (RG-I). In the gastro-intestinal tract the RG-I is only degraded by the action of the colonic microflora. For assessment of potential drug delivery properties, RG-I based microcapsules (~1 μm in diameter) were prepared by an interfacial poly-addition reaction. The cross-linked capsules were loaded with a fluorescent dye (model drug). The capsules showed negligible and very little *in vitro* release when subjected to media simulating gastric and intestinal fluids, respectively. However, upon exposure to a cocktail of commercial RG-I cleaving enzymes, ~ 9 times higher release was observed, demonstrating that the capsules can be opened by enzymatic degradation. The combined results suggest a potential platform for targeted drug delivery in the terminal gastro-intestinal tract.

## Introduction

The uptake of drugs via the oral route is an attractive choice for drug administration as it is easy and convenient. To enhance the therapeutic effect of such oral approaches, several site-specific drug delivery systems, e.g. targeting the colon, have been developed, for example based on pH change, gastro-intestinal (GIT) transit times or bacterial degradation. To formulate drug delivery systems with tailored drug releasing properties, the development of new polymers with unique physicochemical properties is a prerequisite. In general, polysaccharides are interesting as oral based drug carriers, because they are non-toxic and exhibit good biocompatibility and controlled release properties [1]. In particular, pectin is considered an important polysaccharide in this context [2], as it is only degraded by enzymes from the human colonic microflora. Therefore, its suitability for efficient colon drug delivery has been investigated previously. However, the focus in the past has primarily been on solid particles/beads [1, 3] and to a lesser extent on hydrogel particles [4, 5] or oil-core microcapsules [6]. Microcapsules based on rhamnogalacturonan-I (RG-I), which is the focus in the present paper, have not previously been reported.

RG-I belongs to the class of pectic polysaccharides. The pectin macromolecule comprises four major polysaccharides: Rhamnogalaturonan I, Rhamnogalaturonan II, xylogalacturonan and homogalacturonan, of which homogalacturonan (HG) is the most abundant. HG consists of long linear segments of partially methyl-esterified (1–4)-linked α-D- galacturonic acid. It is used as an ingredient (E440) in food industry and often simply referred to as pectin. Drug release from beads of HG has previously been studied [1, 3] and the inherent self-assembly properties of HG, mediated by divalent cations (e.g. zinc and calcium), have made it an attractive material for drug delivery purposes. However, the drug release from beads prepared in this way is time-dependent and due to gradual dissolution/erosion of the beads. In particular, calcium pectinate beads have been shown to disintegrate before reaching the colon [7, 8]. To prevent this, beads can be coated with a silica layer to reduce swelling and delay drug release [7, 8].

RG-I is the second most abundant polysaccharide of the pectin family. It was recently shown that RG-I by itself reduces the proliferation of certain types of colon cancer cells, demonstrating its potential as a bioactive compound [9]. In addition, RG-I has beneficial effects on the fecal microflora [10, 11].

The present study aims to develop a proof-of-concept comprising the preparation of RG-I-polyurea/urethane capsules with aqueous cores and to evaluate their potential use as a platform for encapsulation and delivery of drugs to the colon *via* oral administration. As RG-I can be degraded by the actions of enzymes in the human colon only [10, 11], RG-I based capsules would be expected to have colon-targeted delivery properties. In that case, the RG-I would act as enzymatically degradable “patches/sites” on the capsule wall surface, allowing for efficient release of the capsule content into the colon. This is the first report of using RG-I to generate microcapsules with aqueous cores.

## Materials and Methods

### Materials

2,4-Toluene diisocyanate (TDI), sodium dodecyl sulfate (SDS), sulforhodamine 101 (SR101), cyclohexane, gelatin from bovine skin (type B), 4-bromophenylboronic acid (BPBA), NaCl, tris(hydroxymethyl)aminomethane (Tris), maleic acid, bile extract porcine, pepsin from porcine gastric mucosa, pancreatin from porcine pancreas and calcium chloride dihydrate were purchased from Sigma Aldrich. Gastric lipase from rabbit (RGL) was a gift from Dr. Frédéric Carriére (Laboratory of Enzymology at Interfaces and Physiology of Lipolysis, Centre National de la recherché scientifique, Marseille, France) and phospholipid from egg was received from Lipoid (Ludwigshafen, Germany). GRINSTED® PGPR 90 Kosher (poly(glycerol)poly(ricinoleate)) was a kind gift from Danisco, Denmark. Pectinex Smash XXL (declared enzyme: Pectin lyase), Novozyme 863 (Polygalacturonase), Viscozyme L (Beta-glucanase (endo-1,3(4)-), multi-component plant cell wall degrading enzyme preparation) and Pulpzyme HC (Xylanase (endo-1,4-)) were kind gifts from Novozymes A/S, Denmark.

### Enzymatic extraction of RG-I

**Fig 1:**
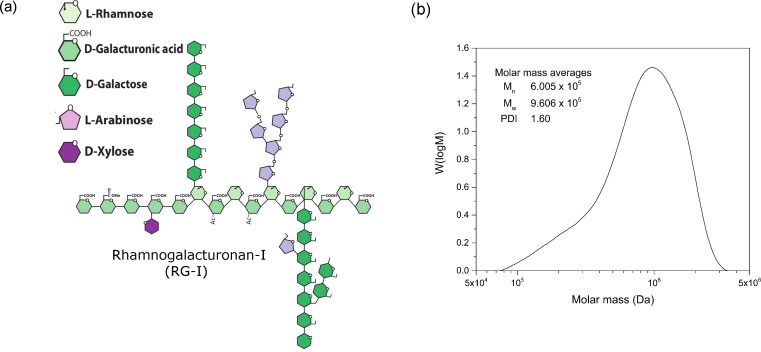
Rhamnogalacturonan-I. (a) The chemical structure of Rhamnogalacturonan-I (b) Molar mass distribution of Rhamnogalacturonan-I.

The RG-I was enzymatically extracted from potato pulp, that is the waste product after starch production. The procedure and characterization are described in detail by Byg et al [12]. Briefly, the pectin macromolecule used in this study contains long linear segments of alternating highly acetylated and partially methyl-esterified α-D- galacturonic acid and L-rhamnose (rhamnogalacturonan I–RG-I) essentially devoid of linear α-D- galacturonic acid (homogalacturonan—HG). Potato RG-I is documented to have a high degree of acetylation (DA) and is found to be in the range of DA 54.7 [13]. The RG-I, produced by the procedure described in Byg et al. [12] was further treated with PG-III (CAE 46195, exo-polygalacturonase, Novozymes, Denmark) until the galacturonic acid:rhamnose (GalA:Rha) ratio was 1.08. A GalA:Rha ratio of 1 is a RG-I molecule devoid of the homogalacturonan tail [12]. The alternating GalA-Rha backbone carries branches of L-arabinans and D-galactans, the former highly branched and the latter predominantly linear, see Fig 1A [14]. In mol% the resulting sugar profile was fucose 2.2; rhamnose 6.7; arabinose 10.5; galactose (Gal) 73.2; glucose 0.0; xylose 0.1; mannose 0.0; galacturonic acid 7.3.

### Microcapsule synthesis

150 mg of RG-I and 21 mg of NaCl was dissolved in 1.31g Milli-Q water. For release experiments, 1 mg of the fluorescent dye sulforhodamine 101 was also added to the aqueous phase. This dye was chosen due to previously reported suitable characteristics, such as pH- and temperature-insensitivity, no reaction with 2,4-Toluene diisocyanate (TDI) [15, 16] and also because the fluorescence signal is comparable in the different simulated media, see S1 Fig. An amount of 7.5 g of a cyclohexane solution, containing 80 mg of poly(glycerol)poly(ricinoleate) (PGPR), was added and the phases were mixed (magnetic stirring) vigorously for 1–1.5 h. The PGPR was used as a surfactant to stabilize the water droplets in cyclohexane. The crude emulsion was subsequently subjected to ultra-sonication (120s, 80% amplitude, pulse 20s, pause 10s, Sonics Sonifier, 750W, 1/2” tip) under ice-cooling. Afterwards, 3 g of cyclohexane, containing 1.4× 10^−3^ mol (0.2 mL) or 1.3×10^−3^ mol (0.19 mL) of TDI was added drop wise. The polyaddition reaction proceeded overnight at 25°C under slow magnetic stirring (400 rpm). A third capsules sample was also prepared for FTIR using a lower amount of cross-linker, 0.44×10^−3^ mol (0.06 mL). However, due to lack of RG-I, the amount of chemicals used in the synthesis of the third capsule type was scaled down; 63 mg RG-I, 8.6 mg NaCl, 0.55 g Milli-Q water, 3.1 g of cyclohexane solution containing 33 mg of PGPR and then 1.3 g of cyclohexane containing 0.18×10^−3^ mol (0.025 mL) TDI.

A 0.5 wt% capsule suspension in Milli-Q water was prepared by mixing the retrieved suspension of capsules in cyclohexane with an 8 mM SDS solution in MilliQ water. The suspension was sonicated at low amplitude 30% for 20s (Sonics Sonifier, 750W, 1/2” tip) to obtain a good dispersion. A low amplitude was used in order to not break the capsules. The solution was left overnight at RT and under magnetic stirring, to evaporate the cyclohexane; in the morning the water content was adjusted in order to account for any water evaporation. A freshly prepared 0.5 wt% capsules suspension in Milli-Q water was used in the release experiments. For FTIR and ^13^C CP/MAS NMR measurements, dry capsule samples were obtained by freezing the suspension of capsules in cyclohexane, followed by freeze-drying.

The encapsulation efficiency was analyzed from the amount of free dye (= not encapsulated) present in the 0.5 wt% capsule suspension in Milli-Q water. Samples were centrifuged at 4000 rpm for 5 min, the supernatant was collected and the amount of free dye was analyzed with fluorescence spectroscopy. The reported value is an average of three measurements.

### In vitro release studies

#### Gastric step

20 mL of concentrated fasted state simulated gastric fluid, SGF (pH ~ 1.64), was added to a thermostated glass vessel (37°C) with overhead stirring and a lid and the pH was adjusted to 1.6 with 5M HCl. 4 mL of the 0.5 wt% capsule suspension and 1 mL of a concentrated RGL dispersion were added (in the experiment without enzymes 1 mL Milli-Q water was added) and a sample (t = 0) was withdrawn. The composition (without of enzyme) of the resulting SGF medium is given in Table 1. The medium also contained pepsin (450 U mL^-1^) and the activity of RGL was 100 U mL^-1^. The composition of the SGF and the enzyme concentrations are based on previous studies [17, 18]. The pH was maintained at pH 1.6 by adjusting with 5M HCl. The gastric step was 1 hour or 3 hours long.

**Table 1:** The composition of the SGF and SIF media (devoid of enzymes).

	NaCl (mM)	Phospholipid (mM)	Bovine Bile Extract (mM)	Tris (mM)	Maleic acid (mM)	CaCl_2_·2H_2_O (mM)
**SGF (25 mL)**	34.2	0.02	0.08	—	—	—
**SIF (40 mL)**	50	0.26	2.95	2	2	1.4

#### Small intestinal step

After one hour of gastric digestion (the 3 hours gastric step was not continued), the composition of the medium was changed to mimic the fasted state simulated intestinal fluid (SIF) by adding a concentrated SIF (pH ~ 6.3). The pH was adjusted with a 0.2 M NaOH solution to pH 6.5. A concentrated dispersion of pancreatic extract in Milli-Q water (5 mL) was added to initiate the intestinal digestion (Pancreatic lipase activity 600 USP U mL^-1^ in the SIF medium) [19, 20]. In experiments without enzyme, Milli-Q water (5 mL) was added instead. The pancreatic extract suspension was prepared as described previously [21]. The composition of the SIF medium is given in Table 1 [22, 23]. During the lipolysis, the pH was maintained at 6.5 by automatic addition of 0.2 M NaOH solution. The intestinal digestion step was conducted for either 1.5 or 95 hours. In 95 hour experiments, in order to avoid massive evaporation, the digesta was transferred (after 26.5 h) to a closed system and incubated at 37°C in a heating cabinet under slow magnetic stirring. The pH was not adjusted in the closed system and dropped only slightly (pH ~ 6.4).

#### Enzyme cocktail step

The combined activities of the enzymes in the enzyme cocktail (Pectinex Smash XXL, Novozyme 863, Viscozyme L and Pulpzyme HC) was chosen based on previous experience and used to ensure the decomposition of RG-I. The enzymes cocktail was added to the SIF medium after 1.5 hours to obtain the following enzyme activities: Pectinex Smash XXL (90 PECTU mL^-1^), Novozyme 863 (15 PGNU mL^-1^), Viscozyme L (0.5 FBG mL^-1^) and Pulpzyme HC (4.6 AXU mL^-1^). The pH was maintained at 6.5 (automatic addition of 0.2 M NaOH). After 21.5 hour a sample was taken out and analyzed. As no additional release of dye could be detected, extra enzyme was added to obtain the additional enzyme activities: Pectinex Smash XXL (625 PECTU mL^-1^), Novozyme 863 (106 PGNU mL^-1^), Viscozyme L (3.4 FBG mL^-1^) and Pulpzyme HC (32 AXU mL^-1^). The suspension was transferred to a closed system and the enzymatic digestion was continued in a heating cabinet at 37°C under slow magnetic stirring for an additional 72 hours.

Samples (500 μL) were withdrawn during the different steps and lipase inhibitor was added (2.5 μL of 1 mM BPBA in methanol) to the withdrawn sample when they contained pancreatin. The samples were centrifuged at 4000 rpm for 5 min and the supernatant was collected and diluted 10 times prior to fluorescence measurements. The release was calculated as the difference between the amounts of dye at time *t* subtracted by the amount of dye present at time zero (t = 0). Volume changes (evaporation, dilution, sample withdrawal) were taken into account in the calculations.

### Characterization

#### Infrared spectroscopy

Infrared spectroscopy spectra were obtained using an ABB MB3000 (ABB, Switzerland). Freeze-dried capsule samples (1–3 mg) were compounded with ca. 110 mg KBr and pressed into pellets. All pellets were further dried prior to measurements. Spectra from 400–4000 cm^-1^ were collected (32 scans, resolution of 1 cm^-1^).

#### Transmission electron microscopy

Transmission electron microscopy images of ultrathin sections of capsules were obtained using a Philips CM 100 TEM (Philips, the Netherlands). Capsules were first embedded in gelatin (10%), cut in small cubes, stained with OsO_4_ and dehydrated in graded series of ethanol and embedded in EPON. The size and wall-thickness of capsules were analyzed using Image J (NIH, U.S.). The average diameter was calculated from 200 measurements and the average capsule wall thickness was calculated from 50 measurements.

#### Fluorescence spectroscopic measurements

Emission spectra were recorded using an excitation/emission wavelength of 587/605 nm with a Spex Fluorolog 3–22 (Jobin Yvon Horiba, France) using an excitation and emission slit width of 2 and 5 nm, respectively. The amount of dye released from the capsules was calculated from a calibration curve that was set up using different concentrations of dye in water.

#### ^13^C CP/MAS NMR measurements

^13^C cross-polarization (CP) MAS NMR experiments were carried out at room temperature on a Bruker Avance 400 spectrometer (Bruker Biospin, Rheinstetten, Germany) operating at Larmor frequencies of 400.13 and 100.62 MHz for ^1^H and ^13^C, respectively, using a double-resonance probe equipped for 4 mm (o.d.) rotors and a spin-rate of 8000 Hz. Spectra were recorded using a recycle delay of 4 s, 4096 scans and a contact time of 1 ms (rf-field strength of 80 kHz for both ^1^H and ^13^C) were utilized for the variable amplitude CP/MAS experiments [24]. High-power TPPM [25] ^1^H decoupling (rf-field strength: 80 kHz) was applied during an acquisition time of 40.9 ms. All spectra were referenced (externally) to the carbonyl resonance of α-glycine at 176.5 ppm.

#### SEC-MALLS analysis

The size-exclusion chromatography (SEC) set-up used was the SECcurity GPC System (PSS Polymer Standard Services GmbH, Mainz, Germany), which consisted of the Agilent 1260 Infinity isocratic pump (G1310B Iso Pump), autosampler (G1329B ALS), refractive index detector (G1362A RID), a multi-angle laser light scattering (MALLS) detector (BIC-MwA7000, Brookhaven Instrument Corp., New York, USA), and a column set including GRAM precolumn (10 μm, 8×50 mm), 100 Å (10 μm, 8×300 mm), and 10000 Å (10 μm, 8×300 mm) analytical columns (PSS Polymer Standard Services GmbH, Mainz, Germany). The columns were thermostated in the SECcurity column compartment at 60°C and the MALLS and RI detectors were thermostated at 45°C. The RG-I sample was directly dissolved in the SEC eluent of dimethyl sulfoxide (DMSO, HPLC grade) with 0.5 wt% LiBr (ReagentPlus) at 60°C. The sample concentration was in the range of 0.4–2 g/L. The injection volume was 100 μl and the flow rate was 0.5 mL/min. The differential refractive index increment (*dn*/*dc*) value for RG-I in DMSO/LiBr 0.5 wt% was measured on-line using the peak area detected by the RI detector. The RI detector constant was determined by the injection of pullulan standards of known molar masses and *dn*/*dc* value provided by PSS Polymer Standard Services GmbH (Mainz, Germany). Data acquisition and processing were carried out by use of WinGPC software (PSS Polymer Standard Services GmbH, Mainz, Germany).

## Results and Discussion

### Rhamnogalacutronan I

The overall architecture of the RG-I resembles that of a bottlebrush with a central backbone of the repeating disaccharide [-4)-α-GalA-(1→2)- α-Rha-(1→-], see Fig 1A. The GalA residues of the backbone are highly acetylated in many species, e.g potato, while the Rha residues carry neutral sugar side-chains (the hairs of the bottle brush, hence the name “hairy regions” coined by Schols et al [26]). The absolute molar mass of RG-I was analyzed by SEC with online MALLS and RI detection. The *dn*/*dc* value for RG-I in DMSO/LiBr 0.5 wt% was 0.0605 mL/g, as determined with the RI detector using a series of known concentrations of the RG-I sample (S2 Fig, Supporting Information). The RG-I used in the present study had a weight average molar mass (M_w_) of 9.606 × 10^5^ Da, a number average molar mass (M_n_) of 6.005 × 10^5^ Da, and a polymer dispersity index (PDI) of 1.60. The molar mass distribution of RG-I is shown in Fig 1B. Approximately, 84% of the RG-I sample has a molar mass in the range from 4.0 × 10^5^ Da to 3.5 × 10^6^ Da. And there is also a fragment (ca. 16%) of smaller RG-I molecules in the range of 8.0 × 10^4^–4.0 × 10^5^ Da.

### RG-I based Microcapsules

**Fig 2:**
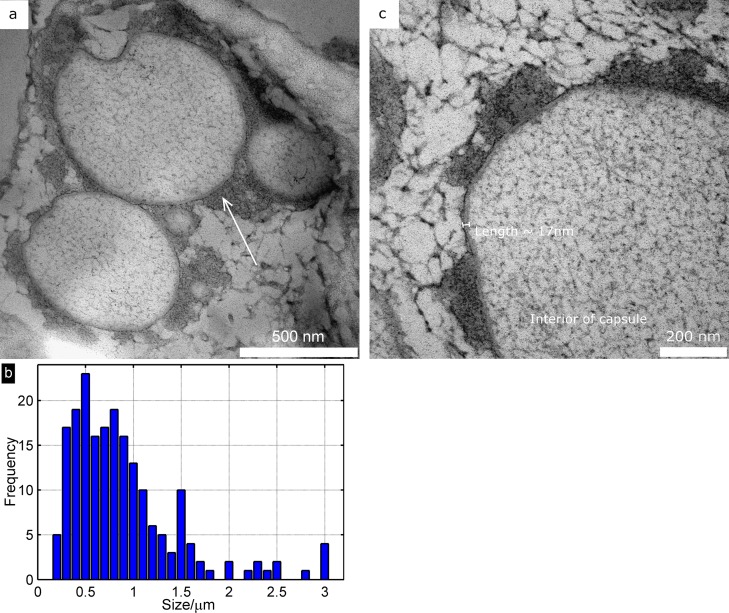
Morphology of capsules. (a) TEM micrographs of the synthesized capsules showing the capsule structure. The arrow points to a darker area, most likely diisocyanate debris from capsule synthesis. (b) Histogram showing the distribution in capsule size. (c) A close-up of a synthesized capsule, showing the cell wall (marked) and the structure of the interior of the capsule.

The RG-I based microcapsules were successfully synthesized using an inverse emulsification technology. The inverse emulsion was formed by mixing (ultra-sonication) an aqueous phase, containing RG-I and sodium chloride, with an oil phase consisting of cyclohexane and polyglycerol polyricinoleate, see the experimental section for details. The capsule wall was formed via a cross-linking process involving the carbohydrate hydroxyls at the oil-water interphase of the aqueous droplets using toluene diisocyanate (TDI) as the cross-linker. TEM micrographs of cross-sections of the synthesized capsules revealed the capsule structure, see Fig 2A. The average size of the capsules was 0.91 ± 0.62 μm and the thickness of the capsule wall was 14.4 ± 2.1 nm (when cross-linked with 1.4×10^−3^ mol TDI). The size distribution was quite broad as shown in Fig 2B. The calculated capsule wall thickness was ~30 nm (calculated as described previously [27] using ρ_urea/urethane_ = 1.25 g cm^-3^ and ρ_RG-I_ = 1.5 g cm^-3^ in the calculations), i.e. the measured capsule wall (14.4 ± 2.1 nm) is thinner than expected (~30 nm) based on the amount of polymer used in the synthesis. This is probably due to that not all polymer participated the capsule wall formation. As an indication of this, in the TEM cross-sections it was possible to see darker areas at the outer capsule shell wall which is most likely diisocyanate debris from capsule synthesis (arrow in Fig 2A). In addition some internal structure, probably due to RG-I, was also visible inside the capsules (see Fig 2C).

**Fig 3:**
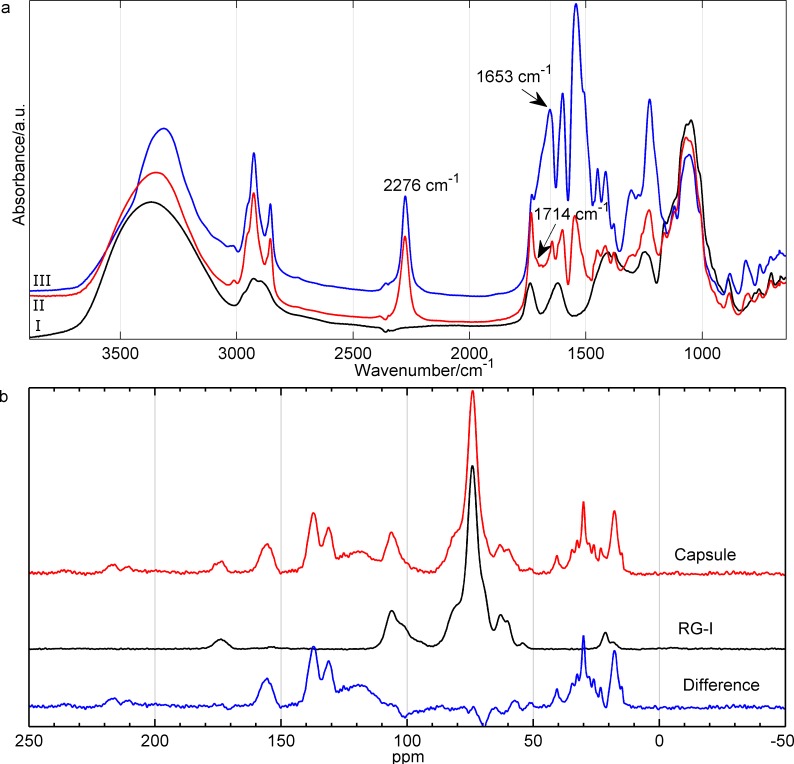
FTIR and ^13^C CP/MAS NMR. (a) FTIR data for neat RG-I (I) and RG-I polyurea/urethane capsules prepared using different amounts of TDI: 0.44×10^−3^ mol (II) and 1.3×10^−3^ mol of TDI (III). (b) ^13^C CP/MAS NMR spectra of pure RG-I and the RG-I polyurea/urethane capsule prepared using 0.44×10^−3^ mol of TDI. The lower NMR spectrum is the difference between the spectrum of the capsule and pure RG-I.

The formed RG-I capsules contained polyurea/urethane cross-links as confirmed by FTIR results presented in Fig 3A. The typical bands for RG-I (Fig 3A) were found at 3370 cm^-1^ (assigned to O-H stretching), 1740 cm^-1^ (stretching vibrations of C = O present in the RG-I backbone) and 1620 cm^-1^ (stretching of C = O in the COO^-^ deprotonated carboxylic functions) [28]. The isocyanate groups of TDI reacted with the hydroxyl groups of RG-I molecules to form urethane groups (˗C = O of urethane at 1714 cm^-1^, shoulder in spectrum II). In addition, the isocyanate groups of TDI were hydrolyzed by water to form amine groups and these further reacted with other isocyanate groups and therefore urea bonds could also be found in the capsule shell (˗C = O of urea at 1653 cm^-1^) [29, 30]. By adding more TDI, the intensity of urea peak increased further, as observed in the FTIR spectra (II) and (III) in Fig 3A, which was rationalized by the enhanced reaction of the isocyanate groups with water resulting in urea formation. Some un-reacted isocyanate groups were also present (2276 cm^-1^, spectra II and III in Fig 3A). These reactive isocyanate groups can be used to functionalize the outer capsule surface or be hydrolyzed to amine groups by transferring the capsules to water, as previously demonstrated for TDI cross-linked starch nano-capsules [16]. The FTIR spectra for the surfactant (PGPR) used in the capsule synthesis is given in the Supporting Information, S3 Fig.

The presence of both urethane and urea linkages in the capsule wall was further supported by solid state ^13^C CP/MAS NMR, see Fig 3B. Previous ^13^C NMR studies report chemical shifts of the carbonyl group in the urethane bond at 158 ppm (diisocyante grafted onto cellulose) [31], at 155.6 ppm (aromatic diisocyante and glycidyl azide polymer) [32] and at 152.75 ppm (polyurea formed by toluene diisocyanate reaction with water) [30]. Note that the latter is similar to the urea formation for the present capsules. The ^13^C CP/MAS NMR spectrum of the capsule in Fig 3B, displays a broad resonance centered at 155 ppm, which is believed to be overlapping resonances originating from carbonyl groups in both urea and urethane linkages in the capsule wall.

The lower NMR spectrum in Fig 3B, is the difference between the spectrum of the capsule and the spectrum of pure RG-I. It was observed that resonances with negative intensity or a dispersive line shape are present around 60, 70 and 101 ppm. This shows that C6 in galactose as well as carbons in the pyranose ring including the anomeric carbons of Gal, Rha or GalA are involved in the linkages between RG-I and TDI.

### Release in vitro

**Fig 4:**
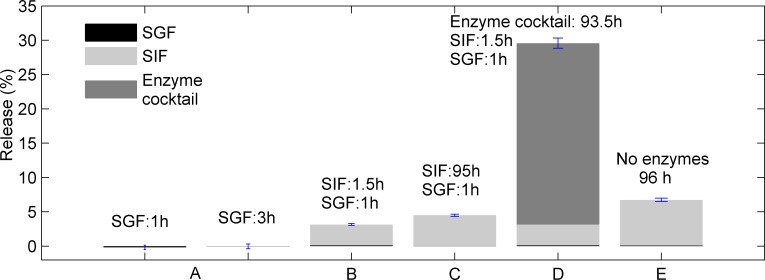
Release of dye from capsules. The release of SR101 (%) from microcapsules (1.4×10^−3^ mol TDI) exposed to simulated media (see Table 1 and experimental section for compositions): A: simulated fasted state gastric fluid (SGF) for 1h or 3h. B: SGF (1h) and simulated fasted state intestinal fluid (SIF, 1.5h). C: SGF (1h) SIF (95h). D: SGF(1h), SIF(1.5h) and Enzyme cocktail (93.5 h) E: SGF without pepsin and gastric lipase (1h) and SIF without pancreatin (95h). The SGF (1h) and SIF (1.5h) experiments in B are the same as those presented in D, but in the D the subsequent exposure to the enzyme cocktail (93.5h) is also included. Note that in all cases (A-E), the release of dye in SGF step is very low. The results are an average of two (B, D) or three (A, C, E) release experiments.

Potential targeted drug delivery properties of the capsules were investigated by studying the release of an encapsulated fluorescent probe (sulforhodamine 101, SR101) serving as a model drug. The encapsulation efficiency was 96.6 ± 2.2%, when using 1.4×10^−3^ mol TDI in the capsule synthesis. The release studies were performed by subjecting the capsules to different sequences of simulated media; see experimental section for details on media compositions. The results of the release studies are presented in Fig 4.

During one hour of exposure to simulated fasted state gastric fluid (SGF, pH: 1.6, 450 U/mL pepsin and 100 U/mL of gastric lipase) negligible release of SR101 from the capsules was observed, see A in Fig 4. The same trend (no release) was observed when increasing the exposure time to 3 hours of SGF, which indicates that the capsules should be stable enough to withstand the acidic conditions and enzymes present in the stomach. The residence time a meal or a dosage form in the stomach of humans is dependent on the composition and caloric output of the ingested bolus, but the average half-emptying time is approximately 60 min [18]. After SGF exposure, some capsules were further exposed to simulated fasted state intestinal fluid (SIF), which was achieved by adding a concentrated solution of amongst others, different salts and surfactants (bile salt and phospholipids) to the SGF suspension containing capsules, and adjusting the pH to 6.5 (see Table 1 for composition). To this medium, pancreatic extract was added (600 U/mL) and the lipolysis was carried out for 1.5 hours at a constant pH. The resulting release was only minor (3.2%) (B in Fig 4) and increasing the exposure time to 95 hours resulted in a release of 4.5% (C in Fig 4). As a comparison, a combined SGF and SIF experiment without pepsin, gastric lipase and pancreatic extract was also conducted (E in Fig 4), with results similar to those in C, suggesting that the observed (low) release in C was due to other mechanisms than enzymatic cleavage of the capsule wall by pepsin, pancreatic enzymes or gastric lipase. These mechanisms are likely to include the presence of surface-active bile salts, inducing release of SR101. More studies are however needed to fully elucidate this aspect. The results in Fig 4 also suggest that the capsules are not degraded by gradual dissolution/erosion mechanisms, as in the case with the pectin beads prepared by ionotropic gelation [1, 3].

One aim of the current paper was to prove that the RG-I capsules can be opened by enzymes that could potentially be secreted by the colonic microbiota. However, the composition of the microbiota and derived enzymes in the colon is the focus of a growing number of research initiatives, due to the strong correlation between health and the microflora [33]. Presently, the polysaccharide hydrolysis in the colon, and the involved microbial enzymes, is poorly understood [34]. Therefore appropriate *in vitro* models for the colonic degradation of RG-I do not exist. Thus, an enzyme cocktail (Pectinex Smash XXL, Novozyme 863, Viscozyme L and Pulpzyme HC) was chosen based on previous experience, and the combined activities were used to ensure the decomposition of the RG-I. The enzymes chosen will act on the side chains as well as the backbone of RG-I. After 93.5 h of incubation, an additional 27% of the SR101 was released (D in Fig 4). This is approximately 9 times more than what was released in the preceding SGF and SIF steps; a total of 3.2% was release in the SGF and SIF step of D in Fig 4. Note that the SGF (1h) and SIF (1.5h) experiments in B are the same as those presented in D. The large release in the presence of the enzyme cocktail, successfully demonstrates that the capsule wall can be enzymatically cleaved despite the presence of polyurea/urethane linkages. By altering the density of the polyurea/urethane network, the enzymatic degradation rate/the release rate of the dye could also potentially be further tailored, as demonstrated previously for lignin-based nano-container systems cross-linked by TDI [35].

## Conclusions

The present study demonstrates that it is possible to synthesize aqueous-core microcapsules utilizing the branched structure of the pectin macromolecule, Rhamnogalacturonan-I (RG-I), in the capsule wall formation. The RG-I based capsules were filled with sulforhodamine 101 (dye) and tested for potential targeted delivery properties in the gastro intestinal tract. No release from the microcapsules was observed upon exposure to simulated fasted state gastric fluid and only a small amount was released during in vitro digestion under the simulated fasted state intestinal conditions. However, upon exposure to a cocktail of four commercial RG-I cleaving enzymes, potentially present in the colon, the capsules were opened. This suggests that the RG-I based capsules have the potential to form an oral delivery platform with colon-targeted drug delivery properties. However, to further confirm this, *in vivo* studies are needed.

## Supporting Information

S1 Fig Fluorescence data for dye in different media. Normalized fluorescence of dye, sulforhodamine 101, subjected to sequence of different simulated intestinal fluid, Gastric step: SGF of pH: 1.6 with 450 U/mL pepsin and 100 U/mL of gastric lipase. Small Intestinal step: SIF with pH: 6.5, pancreatin, 600 U/mL. Enzyme cocktail: pH 6.5, Pectinex Smash XXL (90 PECTU mL^-1^), Novozyme 863 (15 PGNU mL^-1^), Viscozyme L (0.5 FBG mL^-1^) and Pulpzyme HC (4.6 AXU mL^-1^). The fluorescence intensities were normalized by dividing with the average fluorescence intensity obtained for the Gastric step at t = 0. All datapoints were an average of two measurements.
(PDF)

S2 Fig RI peak area plotted vs. the injected mass for five different concentrations of Rhamnogalacturonan-I. The *dn*/*dc* can be obtained from the slope, which depends on the RI detector constant and the *dn*/*dc*.
(PDF)

S3 Fig FTIR data including the surfactant PGPR. (PDF)
