# Supplementary material for: Rhamnogalacturonan-I Based Microcapsules for Targeted Drug Release
Source: PLoS One. 2016 Dec 19;11(12):e0168050. doi: 10.1371/journal.pone.0168050 (PMC5167381; doi:10.1371/journal.pone.0168050)
Supplement: S1 Fig — Normalized fluorescence of dye, sulforhodamine 101, subjected to sequence of different simulated intestinal fluid, Gastric step: SGF of pH: 1.6 with 450 U/mL pepsin and 100 U/mL of gastric lipase. Small Intestinal step: SIF with pH: 6.5, pancreatin, 600 U/mL. Enzyme cocktail: pH 6.5, Pectinex Smash XXL (90 PECTU mL-1), Novozyme 863 (15 PGNU mL-1), Viscozyme L (0.5 FBG mL-1) and Pulpzyme HC (4.6 AXU mL-1). The fluorescence intensities were normalized by dividing with the average fluorescence intensity obtained for the Gastric step at t = 0. All datapoints were an average of two measurements. (PDF) [file pone.0168050.s001.pdf]

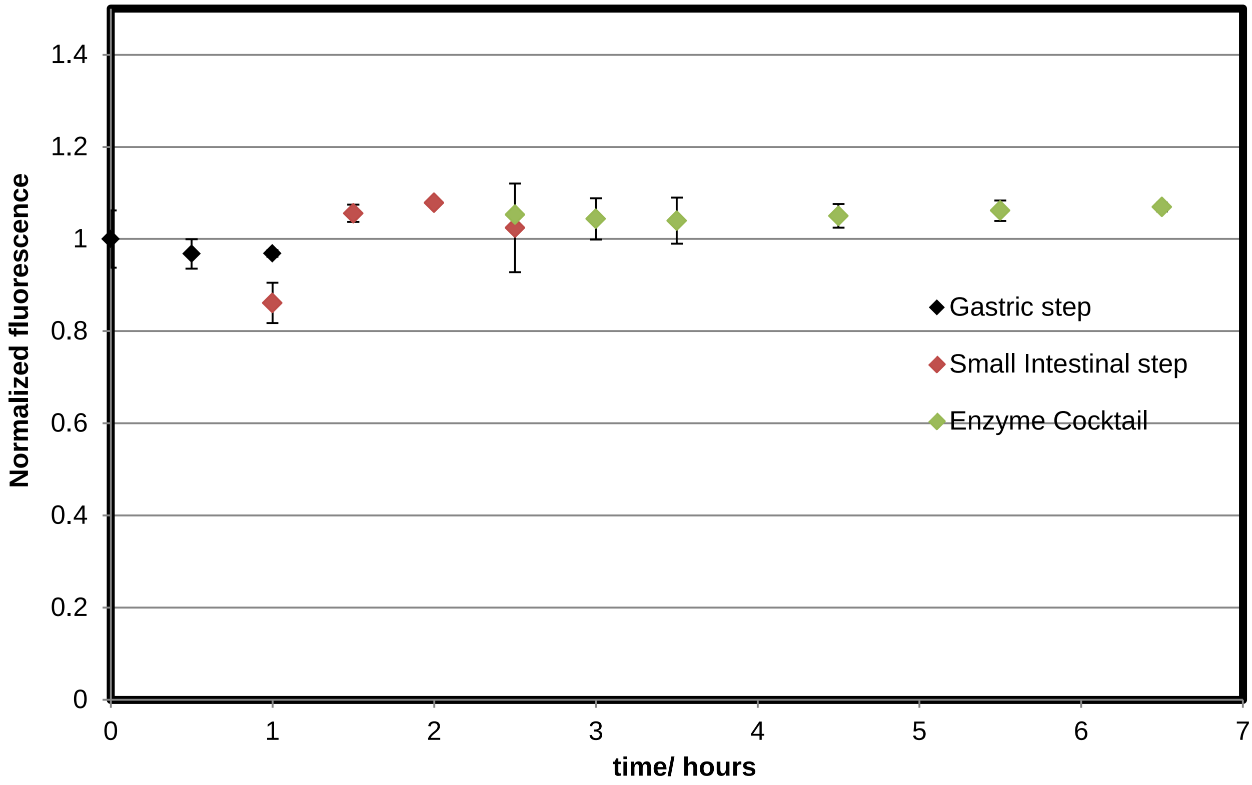

Figure S1. Fluorescence data for dye in different media. Normalized fluorescence of dye, sulforhodamine 101, subjected to sequence of different simulated intestinal fluid, Gastric step: SGF of pH: 1.6 with 450 U/mL pepsin and 100 U/mL of gastric lipase. Small Intestinal step: SIF with pH: 6.5, pancreatin, 600 U/mL. Enzyme cocktail: pH 6.5, Pectinex Smash XXL (90 PECTU mL^{-1}), Novozyme 863 (15 PGNU mL^{-1}), Viscozyme L (0.5 FBG mL^{-1}) and Pulpzyme HC (4.6 AXU mL^{-1}). The fluorescence intensities were normalized by dividing with the average fluorescence intensity obtained for the Gastric step at t=0. All datapoints were an average of two measurements.

The dye, sulforhodamine 101, was subjected to sequence of simulated medium, the experimental setup identical to those described for capsules in *in vitro* release studies; see *in vitro* release studies in the Experimental section. 4 mL of a 5.3×10^{-6} M dye solution in Milli-Q water was added in the experiments. This dye concentration is comparable to that in a 0.5 wt% capsule suspension where all dye has been released from the capsules. As can be observed from Figure S1, the fluorescence is comparable in the different simulated media. The maximum emission wavelength (λ_{em} = 605 nm) did not change in the different media.
