# Supplementary material for: Rhamnogalacturonan-I Based Microcapsules for Targeted Drug Release
Source: PLoS One. 2016 Dec 19;11(12):e0168050. doi: 10.1371/journal.pone.0168050 (PMC5167381; doi:10.1371/journal.pone.0168050)
Supplement: S2 Fig — The dn/dc can be obtained from the slope, which depends on the RI detector constant and the dn/dc. (PDF) [file pone.0168050.s002.pdf]

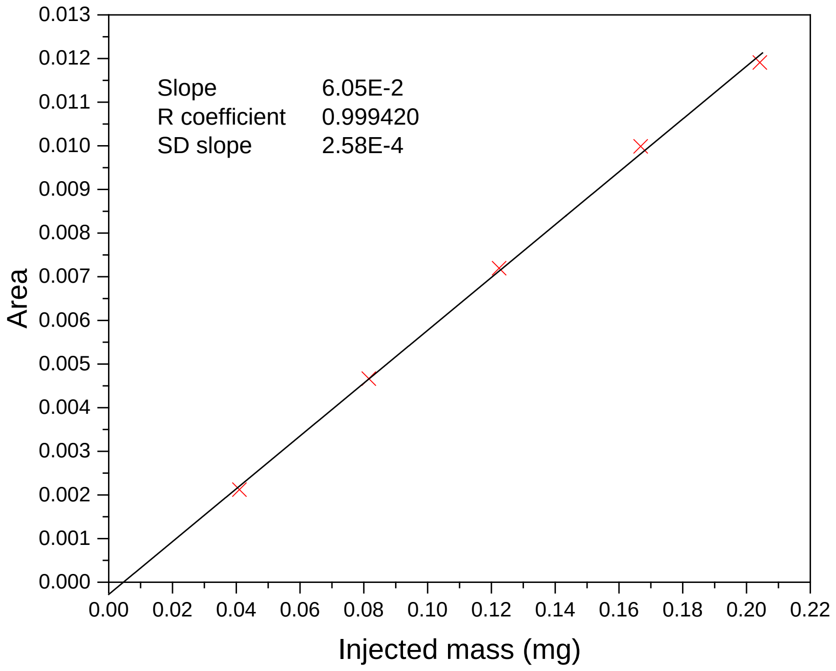

Figure S2. RI peak area plotted vs. the injected mass for five different concentrations of Rhamnogalacturonan-I. The *dn*/*dc* can be obtained from the slope, which depends on the RI detector constant and the *dn*/*dc*.
