# Supplementary figures and images for: Rhamnogalacturonan-I Based Microcapsules for Targeted Drug Release
Source: PLoS One. 2016 Dec 19;11(12):e0168050. doi: 10.1371/journal.pone.0168050 (PMC5167381; doi:10.1371/journal.pone.0168050)

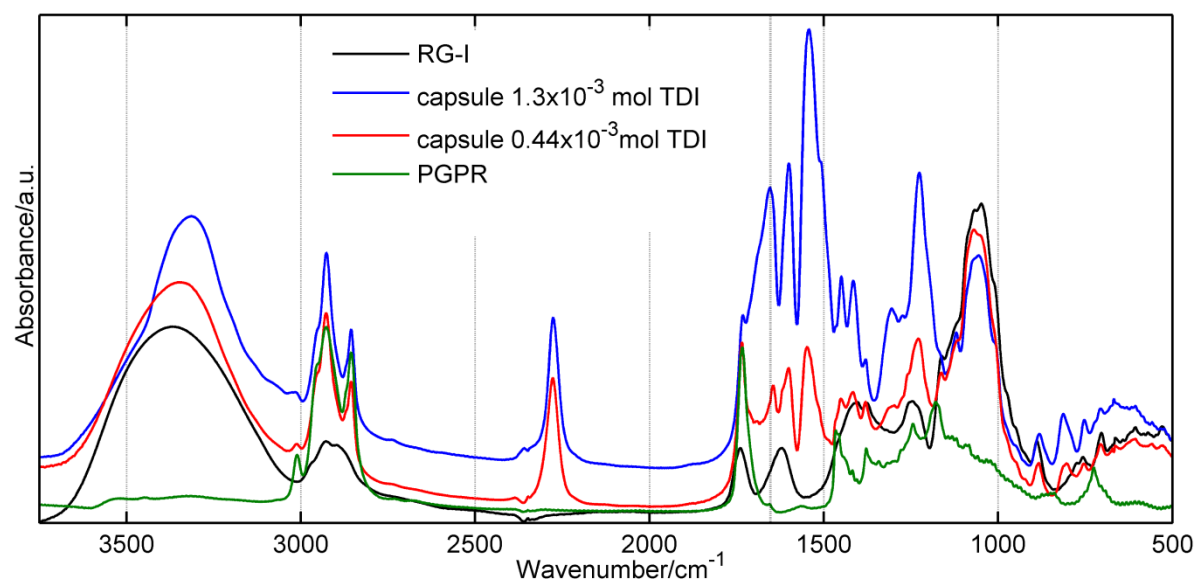

**Figure S3. FTIR data including the surfactant PGPR.**

Supplement: S3 Fig — (PDF) [file pone.0168050.s003.pdf]
